# Effective MRD clearance and long-term survival with CD19 CAR-T in pediatric B-ALL patients with MRD positivity or chemotherapy intolerance

**DOI:** 10.3389/fimmu.2025.1672509

**Published:** 2025-10-06

**Authors:** Yu Wang, Yue-ping Jia, Ai-dong Lu, Le-ping Zhang, Yu-juan Xue, Hui-min Zeng

**Affiliations:** Department of Pediatrics, Peking University People’s Hospital, Peking University, Beijing, China

**Keywords:** chimeric antigen receptor T-cell, B-cell acute lymphoblastic leukemia, minimal residual disease, earlier-line therapy, pharmacokinetics

## Abstract

**Background:**

While CD19-directed chimeric antigen receptor T-cell (CAR-T) therapy demonstrates remarkable efficacy in relapsed/refractory (R/R) ALL, its application in earlier treatment lines requires further investigation. This study aimed to evaluate the efficacy, safety, and cellular kinetics of CD19 CAR-T therapy in pediatric B-cell ALL (B-ALL) patients with minimal residual disease (MRD) positivity or chemotherapy intolerance.

**Methods:**

Between 2017 and 2021, 50 eligible pediatric B-ALL patients (with positive MRD or chemotherapy intolerance) received CD19 CAR-T therapy. Efficacy endpoints included complete remission (CR), MRD-negative CR (MRD-CR), overall survival (OS), and leukemia-free survival (LFS). CAR-T cellular kinetics parameters (C_max_, AUC_0-28d_, persistence) were quantified via qPCR and correlated with clinical outcomes. Safety assessment covered cytokine release syndrome (CRS), immune effector cell-associated neurotoxicity syndrome (ICANS), and infections.

**Results:**

At day 28 post-infusion, the CR and MRD-CR rates were 98% and 96%, respectively. With a median follow-up of 68.7 months, the 5-year OS and LFS rates were 74.9% and 67.8%. Multivariate analysis identified prolonged B-cell aplasia (BCA) duration (HR = 0.969, *p* = 0.021) and female sex (HR = 0.235, *p* = 0.032) as independent protective factors for LFS. Cellular kinetics analysis showed effective *in vivo* expansion in 98% of patients, with a median C_max_ of 30,860 copies/μg DNA and a median time-to-peak of 10.5 days. The MRD-CR group at day 28 exhibited significantly higher C_max_ and AUC_0-28d_ (*p* = 0.017; *p* = 0.029) and superior CAR-T persistence (*p* = 0.030) compared to the non-MRD-CR group. Pre-infusion tumor burden levels did not significantly impact CAR-T expansion or duration. BCA duration positively correlated with CAR-T persistence (r=0.570, *p* < 0.001), but CAR-T expansion parameters (Cmax and AUC0-28d) did not significantly influence BCA. Regarding safety, grade ≥3 CRS occurred in 16% of patients, and ICANS in 10%. Pre-infusion MRD ≥ 10^-3^ was an independent predictor of severe CRS.

**Conclusion:**

CD19 CAR-T therapy demonstrates highly effective MRD clearance and provides long-term survival benefits with a manageable safety profile in pediatric B-ALL patients with MRD positivity or chemotherapy intolerance. Effective CAR-T expansion occurs even at low tumor burdens. These findings support the potential for advancing CAR-T therapy into earlier treatment lines, although its value requires further validation in prospective studies.

## Introduction

Minimal residual disease (MRD) is a critical prognostic biomarker during the treatment of acute lymphoblastic leukemia (ALL) and is closely associated with risk stratification and long-term outcomes (1, 2). The persistence or re-emergence of MRD during therapy often reflects development of chemotherapy resistance and is associated with a heightened risk of relapse (3). Moreover, a subset of patients develops severe treatment-related complications—such as prolonged cytopenias, life-threatening infections, or organ toxicities—that render them unable to tolerate further conventional chemotherapy, thereby creating an urgent need for alternative therapeutic strategies.

In the era of immunotherapy, CAR-T cell therapy has significantly improved outcomes for patients with relapsed or refractory (R/R) ALL, achieving remarkable complete remission (CR) and MRD negativity rate (4). Building on this success, there is growing interest in exploring CAR-T therapy in earlier treatment phases—including MRD-positive settings or as a consolidation strategy—for patients with high-risk features or chemotherapy intolerance. However, several theoretical and practical concerns remain:

1. The low tumor burden in CR may result in suboptimal CAR-T expansion due to reduced antigen exposure.
2. Clinical experience and robust efficacy data regarding CAR-T use in preemptive or early-line settings are still limited.

Emerging evidence from studies in large B-cell lymphoma (LBCL) supports the feasibility and efficacy of CAR-T even among patients achieving CR prior to infusion (5–7). The ZUMA-12 trial, for example, reported high response rates and durable survival in high-risk LBCL patients receiving axicabtagene ciloleucel (axi-cel) as first-line treatment (8). In ALL, although clinical experience is accumulating, dedicated studies focusing on CAR-T therapy in non-bulky, MRD-positive, or chemotherapy-intolerant patients remain limited. Moreover, the cellular pharmacokinetics and expansion dynamics of CAR-T products in the setting of low disease burden are still poorly characterized.

This study was designed to address these unresolved questions by evaluating the efficacy, safety, and cellular kinetics of CD19 CAR-T therapy in a well-defined, homogeneous cohort of pediatric B-ALL patients with either MRD positivity or chemotherapy intolerance. We provide long-term follow-up data and detailed kinetic analyses that distinguish our study from previous reports, offering new insights into the potential application of CAR-T therapy in earlier lines of treatment.

## Materials and methods

### Patients

Between June 2017 and March 2021, 50 pediatric B-ALL patients receiving CD19 CAR-T therapy were enrolled in the trial at our center. Inclusion criteria comprised: (1) Persistent or recurrent MRD positivity during conventional chemotherapy, immunotherapy, or hematopoietic stem cell transplantation (HSCT); (2) Inability to continue conventional chemotherapy due to serious treatment-related complications (termed chemotherapy intolerance) was objectively defined by the occurrence of any of the following events attributable to chemotherapy: (i) prolonged severe myelosuppression (neutrophil count < 0.5 × 10^9^/L or platelet count < 20 × 10^9^/L) lasting >4 weeks despite adequate supportive care; (ii) life-threatening infection (e.g., sepsis, fungal pneumonia) during neutropenic phases; (iii) grade ≥3 non-hematologic organ toxicity (e.g., cardiotoxicity, hepatotoxicity, neurotoxicity) according to common terminology criteria for adverse events (CTCAE) v5.0; or (iv) recurrent febrile neutropenia episodes necessitating hospitalization. The study was approved by the Ethics Committee of Peking University People’s Hospital and conducted in accordance with the Declaration of Helsinki. Written informed consent was obtained from each patient’s parents, or their guardians.

### Anti-CD19 CAR T-cell manufacture and lymphodepletion regimen

CAR-T cells were derived from peripheral blood mononuclear cells (PBMCs) collected from patient/donor leukapheresis. For patients without HSCT history, PBMCs were autologous. For those with prior HSCT, PBMCs were donor-derived. PBMCs were stimulated with dynabeads coated with anti-CD3 and anti-CD28 mAbs (Thermo Fisher Scientific). Activated T cells were transduced with lentiviral vector encoding the anti-CD19 CAR construct consisting of CD19 recognition domain, transmembrane link domain, 4-1BB intracellular domain, CD3ζ intracellular domain. After lentiviral transduction, the CAR-T-19 cells were cultured in medium supplemented with 500 IU/ml IL-2 at 37 °C/5% CO_2_ for approximately 5 to 11 days to obtain sufficient cells for infusion. The final product, CAR-T-19, was prepared by diluting CAR-T cells with 2% human albumin-containing saline. The main component of the final product consisted of CAR-positive CAR-T-19 cells, with a small proportion of natural killer cells. The cell preparation process met the product quality and activity requirements outlined in the guidelines for CAR-T cell therapy products. The final product exhibited a cell activity of not less than 70% and remained effective within 12 hours at the temperature ranging from 10°C to 25°C.

From Day -5 to -3 before infusion, the LD regimen was administered: Fludarabine 30 mg/m² and Cyclophosphamide 300 mg/m² daily for 3 days.

### Diagnostic definitions, MRD detection, efficacy and adverse reaction assessment

1. Cytogenetic risk stratification: Based on the National Comprehensive Cancer Network (NCCN) 2024 risk stratification criteria (9).
2. MRD re-emergence: Redetection of previously negative MRD on ≥2 consecutive occasions (≥2 weeks apart) with bone marrow blasts <5% and no intervening targeted therapy. MRD positivity defined as: flow cytometry (FCM)-MRD ≥0.01% and/or qPCR detection of previously positive fusion genes.
3. MRD detection: MRD was assessed via FCM (sensitivity 10^-4^) on day 28 post-infusion, and then subsequently at regular intervals typically every 2 months for the first year, every 3 months from year 2 to 3, and every 6 months from year 3 to 5, with a total monitoring duration of 5 years, or as clinically indicated upon suspicion of relapse. For patients who underwent HSCT after CAR-T therapy, MRD was assessed at 0, 1, 2, 3, 4.5, 6, 9, and 12 months after HSCT, and then every 6 months thereafter until 5 years post-transplantation.
4. B-cell aplasia (BCA): Peripheral blood or bone marrow analysis showing either B cells ≤1% of white blood cells or B cells ≤3% of lymphocytes (10).
5. CRS and ICANS were graded according to the 2018 American Society for Blood and Marrow Transplantation (ASBMT) guidelines (11).

### Pharmacokinetics of CAR-T cells

CAR-T cell pharmacokinetics were characterized through quantitative analysis of individual concentration-time profiles in peripheral blood, employing non-compartmental analysis (NCA) methodology. Circulating CAR-T cell levels were quantified via quantitative PCR (qPCR) using transgene-specific primers, with results expressed as copy numbers per microgram (μg) of genomic DNA. PK monitoring was performed pre- and post-infusion at 7 days, 14 days, 28 days, 12 weeks, 26 weeks, 39 weeks, 54 weeks, and 104 weeks post-infusion. From year 3 to year 5 after infusion, monitoring was performed every 6 months until becoming undetectable. Monitoring was terminated when peripheral blood CAR-T levels fell below the lower limit of quantification (LLOQ; 100 transgene copies/μg genomic DNA) on two consecutive assessments. Key PK parameters include:

C_max_: Peak level of genetically modified cells *in vivo* post-infusion.
T_last_: Duration of detectable CAR-T transgene in peripheral blood.
T_max_: Time to reach peak CAR-T cell expansion.
AUC_0–28d_: Area under the curve representing total transgene exposure during the expansion phase (Days 0–28).

### Statistical analyses

The last follow-up date was June 30, 2025. OS was calculated from the date of CAR-T cell infusion to death from any cause or last follow-up, whichever occurred first. Similarly, leukemia-free survival (LFS) was measured from CAR-T infusion to relapse, death, or last follow-up, whichever came first. Data were not censored when new therapy (including chemotherapy or allo-HSCT) was initiated in the absence of active disease. Continuous variables were expressed as mean ± standard deviation for normally distributed data or median (range) for non-normally distributed data. Categorical variables were presented as counts (percentages). Categorical variables were compared using χ² or Fisher’s exact tests, while continuous variables were analyzed with t-tests (normally distributed data) or Mann-Whitney U tests (non-normally distributed data). Survival analyses employed Kaplan-Meier curves with log-rank tests, and multivariable analysis used Cox proportional hazards models. Multivariable logistic regression identified risk factors for adverse events. Statistical significance was defined as *p* < 0.05. Cumulative incidence of relapse (CIR) analysis was performed using R software (Bell Labs, New Providence, NJ, USA), other statistical analyses used SPSS 26.0 (SPSS Inc., Chicago, IL, USA), and figures were generated with GraphPad Prism 9.5.1 (GraphPad Software, LLC, USA).

## Results

### Patient characteristics

A total of 50 consecutive B-ALL patients with MRD positivity or chemotherapy intolerance who underwent CAR T-cell therapy at Peking University People’s Hospital were included in this study. Baseline characteristics are summarized in **Supplementary Table S1**. Enrolled patients comprised 7 cases (14%) intolerant to chemotherapy due to treatment-related severe complications, 41 cases (82%) with MRD re-emergence during therapy, and 2 cases (4%) persistently MRD-positive after achieving remission with intensive chemotherapy. The median follow-up time post-infusion was 68.7 months (range: 5.9–97.5). Patients received a median CAR-T dose of 3.68 × 10^6^ cells/kg (range: 0.05–6.33 × 10^6^), with a median interval from diagnosis to CAR-T infusion of 15.8 months (range: 1.5–132.0).

### Treatment outcomes

Forty-nine patients (98%) achieved CR and/or complete remission with incomplete hematologic recovery (CRi), including 48 (96%) attaining MRD-negative CR/CRi at 28 days after CD19 CAR-T cell infusion. The 5-year OS rate was 74.9% [95% Confidence Interval (CI), 64.8–85.1%], and the 5-year LFS rate was 67.8% (95% CI, 57.5–78.0%). Fifteen patients (30%) experienced relapse post-infusion. The median time from infusion to relapse was 12.0 months (range: 0.5–38.4 months). Among them, 4 patients experienced CD19-negative relapse with a median relapse time of 5.0 months, while the other 11 cases were CD19-positive relapse with a median relapse time of 15.7 months. Based on CAR-T persistence, patient preference, and donor availability, 32 patients bridged to allo-HSCT after CAR-T infusion, with a median bridging interval of 2.6 months (range: 1.3–9.0).

Univariate analysis results are presented in **Supplementary Table S2**. All variables with *p* values <0.2 were included in subsequent multivariate Cox regression analyses. In the multivariate analysis, longer BCA duration (HR, 0.969; 95% CI, 0.944–0.995; *p* = 0.021) and female patients (HR, 0.235; 95% CI, 0.062–0.884; *p* = 0.032) were associated with longer LFS (**Table 1A**). While absence of prior transplantation and no ICANS post-infusion showed potential protective effects on OS, these associations were not statistically significant (**Table 1B**). Notable, no significant association between OS/LFS and the following variables: patient age, genetic risk factors, CAR-T history, pre-infusion MRD status, CAR-T cell dose, severe CRS, bridging to HSCT, or CD19 CAR-T cell expansion.

**Table 1:** Multivariable analysis of LFS (A) and OS (B) for subgroups.

(A)			
Covariate	LFS		
	HR	95%CI	*p*
BCA	0.969	0.944,0.995	0.021
Female	0.235	0.062,0.884	0.032
MRD-CR on Day 28	0.910	0.148,5.578	0.919

(B)			
Covariate	OS		
	HR	95%CI	*p*
No CAR-T history	0.128	0.015,1.059	0.057
Grade 4 neutropenia	1.023	0.527,1.983	0.947
CRS(≥3)	1.122	0.203, 6.289	0.895
ICANS (≥1)	6.036	0.868,42.464	0.071
HSCT after CAR-T	0.514	0.108,2.446	0.403
BCA	0.990	0.971,1.009	0.286

MRD, minimal residual disease; HSCT, hematopoietic stem cell transplantation; CRS, cytokine release syndrome; ICANS, immune effector cell-associated neurotoxicity syndrome; CR, complete remission; CAR-T, Chimeric antigen receptor-T lymphocytes; LFS, leukemia-free survival; OS, overall survival; HR, hazards ratio; CI, confidence interval; BCA, B-cell aplasia.

**Figure 1:**
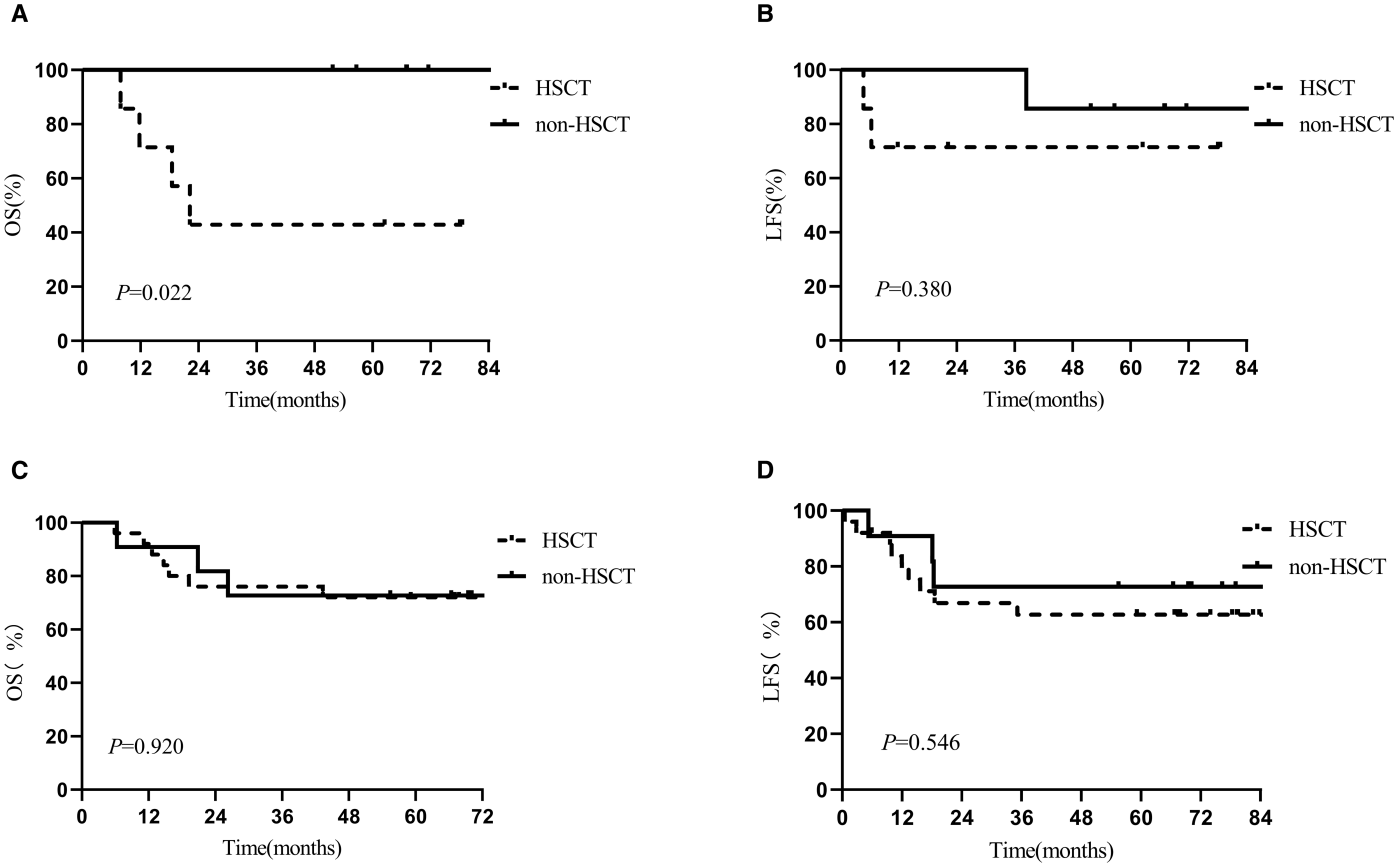
Kaplan-Meier estimates of 5-year outcomes in the HSCT and non-HSCT arms in different MRD group. **(A)** OS in MRD<10^-4^, **(B)** LFS in MRD<10^-4^, **(C)** OS in MRD≥10^-4^, **(D)** LFS in MRD≥10^-4^.

To evaluate the effect of post-infusion bridging transplantation across different pre-CAR-T tumor burdens, bone marrow assessments were conducted at two timepoints: prior to LD (pre-LD) and post-LD. Stratified Kaplan-Meier analysis demonstrated that in patients with low tumor burden (pre-LD MRD < 10^-4^), bridging transplantation was associated with significantly inferior OS (median OS: 22.2 vs. 67.0 months; *p* = 0.022) (**Figure 1**) and higher non-relapse mortality (NRM: 15.4% ± 1.1% vs. 0%). The detrimental impact of transplantation on OS persisted in patients with post-LD MRD <10^-4^ (median OS: 43.3 vs. 68.3 months; *p* = 0.031). However, bridging transplantation showed no significant effect on LFS across all tumor burden strata (**Figure 1**). Subgroup analyses indicated that patients with high tumor burden (MRD ≥ 10^-2^) in the non-transplant group showed a trend toward reduced OS and LFS, though statistical significance was not reached, likely due to limited sample size (**Supplementary Figure S1**). Besides, no statistically significant differences were detected between different genetic risk groups (high-risk vs. low/intermediate-risk) (**Supplementary Figure S2**).

### Toxicity

**Supplementary Table S3** summarizes adverse events within 4 weeks post-CD19 CAR-T infusion. Grade 4 neutropenia was observed in 48% (24/50). The median nadir absolute neutrophil count was 0.53 × 10^9^/L (range: 0.05–1.45 × 10^9^/L). One patient (2%), who had a pre-LD MRD level of 3.22%, developed intermittent high fever, cytopenia, hyperferritinemia, hypofibrinogenemia, and elevated transaminases shortly after infusion, meeting the diagnostic criteria for hemophagocytic lymphohistiocytosis (HLH). Initial treatment with tocilizumab and glucocorticoids was ineffective in controlling the inflammatory response; ultimately, plasma exchange led to symptom resolution. Within the first 4 weeks post-infusion, a total of 9 patients (18.0%) experienced infections of any grade. These included bacterial bloodstream infections (n=4), viral reactivations (CMV n=2, EBV n=1), and invasive fungal infections (n=2). CRS and severe CRS (≥ grade 3) occurred in 68.0% and 16.0% of patients, respectively. ICANS incidence was 10.0%. Fourteen patients (28.0%) received tocilizumab, and 7 (14.0%) required corticosteroid therapy after infusion. None of the 5 patients with prior HSCT developed graft-versus-host disease (GVHD) post-infusion.

Univariate analysis (**Table 2**, **Supplementary Table S4**) identified that patients with high pre-infusion tumor burden (defined as MRD ≥10^-3^ either pre- or post-LD) had significantly increased risks of ICANS and severe CRS. CD19 CAR-T cell dose also influenced CRS severity: patients receiving doses below the median threshold (3.66 × 10^6^ cells/kg) exhibited reduced CRS risk (*p* = 0.049). Both pre- and post-LD MRD levels retained independent predictive value for severe CRS when incorporated into the multivariate analysis separately. Multivariate analysis for ICANS was precluded by the limited number of events (n=5).

**Table 2:** Univariate screening (P<0.2) and multivariate analysis of clinical factors associated with grade ≥3 CRS.

Clinical factors	Univariate analysis		Multivariate analysis			
	OR (95%CI)	*p*	OR(95%CI) ^#^	*p* ^#^	OR(95%CI) ^*^	*p* ^*^
female	0.544(0.361-0.821)	0.055	0.364(0.030-4.330)	0.424	0.185(0.017-62.070)	0.171
No HSCT history	0.286(0.056-1.446)	0.176	0.391(0.031-4.874)	0.465	0.135(0.007-2.496)	0.178
Pre-LD MRD<10^-3^	0.381(0.231-0.629)	0.007	0.067(0.006-0.725)	0.026		
Post-LD MRD<10^-3^	0.349(0.183-0.667)	0.013			0.067(0.006-0.711)	0.025
CAR-T dose<3.68×10^6^ cells/kg	0.490(0.317-0.758)	0.049	0.089(0.008-0.968)	0.047	0.138(0.013-1.513)	0.105

MRD, minimal residual disease; HSCT, hematopoietic stem cell transplantation; LD, lymphodepletion; OR, odds ratio.

#Multivariate Model 1 OR (95%CI) and *p*-value.

*Multivariate Model 2 OR (95%CI) and *p*-value.

Post-infusion, 78% of patients (39/50) developed elevated interleukin-6 (IL-6) levels, with a median peak concentration of 33.3 pg/mL (range 7.6 to >5000) occurring at a median of 8 days (range 1-22). Elevated IL-6 levels showed strong predictive capacity for both severe CRS (OR = 5.250) and ICANS (OR = 9.000) (*p* < 0.001 for both), suggesting its utility as an early-warning biomarker for CAR-T-associated toxicities.

### PK of CAR-T cells

Robust *in vivo* expansion of CAR-T cells was achieved in 98% of patients (49/50). Pharmacokinetic evaluation demonstrated median time-to-peak expansion (T_max_) at 10.5 days (range 4-21), with median peak CAR-T cell levels (C_max_) reaching 30,860 copies/μg genomic DNA (range 1,602-1,890,000). Significant positive correlations were observed between CAR-T expansion kinetics and *in vivo* persistence (T_last_) (C_max_: r = 0.349, *p* = 0.016 and AUC_0–28d_: r = 0.313, *p* = 0.032) (**Supplementary Figure S3**). Responders at day 28 demonstrated significantly enhanced expansion kinetics compared to non-responders, with MRD-negative CR patients (n=48) exhibiting higher geometric mean C_max_ (319,016 vs. 863 copies/μg DNA; *p* = 0.017), greater exposure (AUC_0-28d_, *p* = 0.029), and improved CAR-T cell persistence (*p* = 0.030).

Patients receiving corticosteroids post-infusion showed higher C_max_ (*p* = 0.017) and AUC_0–28d_ (*p* = 0.012), potentially reflecting more pronounced inflammatory responses. However, corticosteroid use had no significant impact on CAR-T persistence (*p* = 0.502). Although patients with high tumor burden (**Supplementary Figure S4**) and those experiencing severe CRS/ICANS (**Table 3**) showed upward trends in C_max_ and AUC_0–28d_, these differences did not reach statistical significance.

**Table 3:** Clinical determinants of PB CD19 CAR-T kinetics (qPCR).

Characteristics	N, n(%)	Median C_max_ (copies/ug DNA)	*p*	Median AUC_0-28d_ (copies/ug DNA×d)	*p*	Median T_last_, d	*p*
Age, y			0.461		0.361		0.406
0-10	29	30490		147035		30	
>10	21	33000		247033		46	
Sex			0.823		0.592		0.381
Male	27	32892		247033		48	
Female	23	25103		162532		30	
HSCT history			0.357		0.447		0.368
Yes	5	36226		310429		60	
No	45	25103		190746		32	
CAR-T history^#^			0.373		0.488		0.341
Yes	2	78247		799473		30	
No	48	30645		203017		40	
Pre-LD MRD			0.342		0.713		0.368
<10^-4^	14	27796		183651		29	
≥10^-4^	36	31910		231161		46	
Pre-LD MRD			0.438		0.644		0.310
<10^-3^	29	25103		176556		30	
≥10^-3^	21	32901		247033		45	
Pre-LD MRD			0.133		0.180		0.582
<10^-2^	45	30490		190746		37	
≥10^-2^	5	81330		310429		60	
Post-LD MRD			0.502		0.661		0.940
<10^-4^	23	30920		190746		38	
≥10^-4^	27	30800		247033		40	
Post-LD MRD			0.084		0.108		0.195
<10^-3^	33	30490		176556		30	
≥10^-3^	17	82300		274715		54	
Post-LD MRD			0.198		0.217		0.948
<10^-2^	47	30800		190746		37	
≥10^-2^	3	294000		1436164		50	
CAR-T dose			0.720		0.884		0.873
≥3.68×10^6^ cells/kg	25	23519		190746		47	
<3.68×10^6^ cells/kg	25	30920		215288		35	
CRS			0.354		0.234		0.857
0-2	42	30645		183651		32	
3-4	8	100799		873296		50	
ICANS			0.374		0.410		0.782
No	45	30800		190746		37	
Yes	5	135522		586499		50	
Grade 4 neutropenia			0.214		0.214		0.623
Yes	24	35463		267850		32	
No	26	27796		161795		46	
Tocilizumab			0.167		0.054		0.766
Yes	14	70631		308252		50	
No	36	27951		169544		32	
Steroids			0.017		0.012		0.502
Yes	7	135522		1454188		54	
No	43	25103		162532		35	
MRD-CR on Day 28			0.017		0.029		0.030
Yes	48	31906		227218		45	
No	2	863		16072		24	

MRD, minimal residual disease; HSCT, hematopoietic stem cell transplantation; CRS, cytokine release syndrome; ICANS, immune effector cell-associated neurotoxicity syndrome; CR, complete remission; CAR-T, Chimeric antigen receptor-T lymphocytes; LD, lymphodepletion; PB, peripheral blood; qPCR, quantitative real-time polymerase chain reaction.

# Patients with CAR-T history were treated only CD19 CAR before joining this clinical trial.

**Figure 2:**
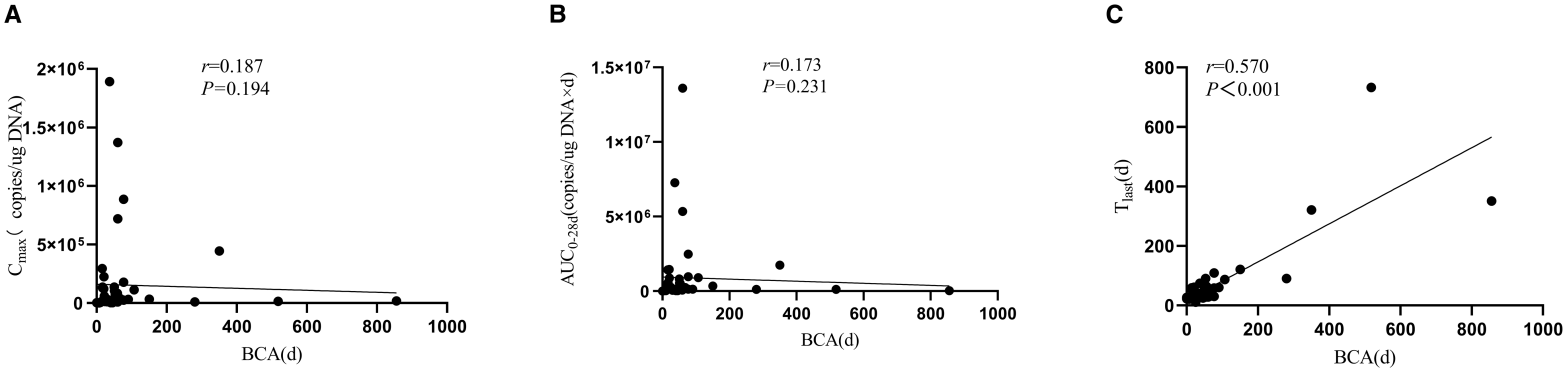
Association between B-cell aplasia duration and CAR-T-19 pharmacokinetic parameters. **(A)** C_max_, **(B)** AUC_0-28d_, **(C)** T_last._.

BCA developed in 96% of patients (48/50), with a median onset of 1–20 days and a median duration of 59 days. Notably, BCA duration exhibited a strong positive correlation with CAR-T persistence (r = 0.570, *p* < 0.001), but showed no significant association with CAR-T expansion parameters (C_max_ and AUC_0–28d_; **Figure 2**).

## Discussion

Accumulating clinical evidence supports the therapeutic efficacy of CAR-T therapy in R/R B-ALL. The global ELIANA trial demonstrated an 81% overall response rate (ORR) with tisagenlecleucel in pediatric/adolescent patients, accompanied by 5-year event-free survival (EFS) and OS rates of 42% and 55%, respectively (12). Importantly, multiple studies consistently identify MRD status as a critical prognostic factor, wherein MRD positivity correlates with significantly elevated risks of relapse and mortality (3, 13). Our institutional data corroborate these findings: patients exhibiting persistent or recurrent MRD during therapy achieved a 5-year OS of 49.8% ± 4.3%, with a CIR of 60.2% ± 4.3% (14).

Building upon this clinical foundation, we conducted a retrospective study of 50 pediatric B-ALL patients with persistent MRD positivity or chemotherapy intolerance who received CD19 CAR-T therapy. Our results demonstrated robust efficacy, with a Day 28 ORR of 98%, MRD-negative CR rate of 96%, and 5-year OS of 74.9%. One important reason about the favorable long-term outcomes compared to historical studies in R/R ALL is that our cohort consisted of patients with MRD positivity or chemotherapy intolerance, not overt, bulky relapsed/refractory disease. This inherently selects for a patient population with a lower disease burden and potentially less aggressive disease biology, including reduced genetic evolution and chemoresistance. And these findings corroborate the ZUMA-3 trial outcomes, wherein earlier CAR-T intervention conferred superior survival benefits: patients receiving CAR-T after one prior therapy line achieved 80% 1-year OS versus 69% in heavily pretreated patients (≥2 prior lines) (15). Growing evidence supports CAR-T integration into earlier-line settings: ① A single-center trial of CAR-T consolidation in elderly B-ALL patients showed durable efficacy; ②Huang et al. reported 2-year OS rates of 92% when combining CAR-T with dasatinib in 29 treatment-naive Ph+ ALL patients (16), and ③ An MD Anderson Cancer Center (MDACC) real-world study using brexucabtagene autoleucel (brexu-cel) as consolidation therapy in 52 adult B-ALL patients demonstrated 1-year relapse-free survival (RFS) and OS rates of 65% and 90%, respectively. Collectively, these data underscore CAR-T’s potential in treatment-naive or minimally pretreated populations. Blinatumomab has also shown efficacy in patients with MRD-positive B-ALL, achieving MRD-negative responses in 97% of cases in the era of immunotherapy (17). In contrast to continuous infusion-based therapies such as blinatumomab, which often require extended hospitalization or the use of portable pumps, CAR-T therapy offers the advantage of a single administration with potential for sustained activity *in vivo*. The decision between these immunotherapeutic strategies, however, depends on multiple factors, including urgency of response, institutional experience, drug availability, cost, as well as patient and clinician preferences. Further prospective studies are warranted to directly compare the clinical roles of different immunotherapies—such as CAR-T versus blinatumomab—in earlier-line treatment of ALL patients.

Advancing CAR-T therapy to earlier treatment lines offers significant advantages. First, patients in earlier disease stages typically exhibit superior performance status and fewer comorbidities, enhancing tolerance to CAR-T infusion and improving safety outcomes-as evidenced by the 10% ICANS and 16% severe CRS rates in this cohort (18). Second, collecting lymphocytes during earlier disease stages circumvents chemotherapy-induced impairment of T-cell viability and function, thereby increasing the likelihood of generating products enriched with stem cell memory T cells (TSCM) or central memory T cells (TCM) that may enhance therapeutic efficacy (19). Although phenotypic characterization of infused CAR-T cells was not performed in our study, the favorable 5-year OS (74.9%) indirectly supports the clinical benefits of early intervention in B-ALL. Consequently, implementing CAR-T therapy earlier (e.g., at MRD positivity or as earlier-line treatment) reduces exposure to adverse prognostic factors such as high tumor burden and cumulative therapy toxicity (20), thus optimizing both efficacy and safety profiles.

In this cohort, 64.0% of patients underwent HSCT following CD19 CAR-T therapy. The role of post-CAR-T HSCT consolidation remains controversial: while a Phase I trial from Seattle Children’s Hospital reported significantly lower relapse rates with HSCT consolidation versus CAR-T monotherapy (18% vs. 55%) (21), the ELANA trial found no significant impact of HSCT on OS (22). Our data demonstrate that for early-phase B-ALL patients without active relapse, bridging to HSCT post-CAR-T conferred no significant OS or LFS benefit. Subgroup analyses further revealed no significant advantage across pre-infusion MRD strata or genetic risk groups. Notably, HSCT was associated with increased NRM attributable to transplant-related complications-particularly in the MRD < 10^-4^ group (NRM: 15.4% ± 1.1%)-which may compromise overall survival in transplanted patients, consistent with prior reports (23, 24). Consequently, for patients with low pre-infusion tumor burden (e.g., MRD < 10^-4^), HSCT should be deferred. Subsequent clinical management can be guided by monitoring peripheral BCA, CAR-T persistence, and MRD assessments (25–27). For high tumor burden patients (MRD ≥10^-2^), however, HSCT may provide clinical benefit despite the lack of statistical significance in our subgroup analysis, likely due to limited sample size. Furthermore, the high rate of subsequent HSCT bridging (64%) in our cohort, while reflecting real-world clinical practice, represents a key study limitation. The relatively small size of the non-HSCT group reduces the statistical power to evaluate the standalone efficacy of CAR-T therapy and may introduce selection bias, as the decision to proceed to transplant was non-randomized and influenced by factors such as donor availability and physician preference.

Current evidence indicates that sufficient antigenic stimulation is required to activate CAR-T cells and drive clonal expansion (28)—a prerequisite for antitumor efficacy. Consequently, evaluating CD19 CAR-T expansion kinetics in CR patients is imperative. To address this, we investigated key determinants of cellular kinetics governing CAR-T expansion and persistence in CR patients. Our data demonstrate rapid CAR-T cell expansion to peak levels at a median of 10.5 days post-infusion, consistent with established expansion models where maximal activity occurs within two weeks (29, 30). Notably, the *in vivo* proliferative capacity of CAR-T cells generates PK profiles characteristically distinct from conventional therapeutics. Infusion dose exhibited no significant impact on expansion magnitude or durability, aligning with prior reports (10). Furthermore, varying pre-infusion MRD levels similarly had no significant effect on expansion kinetics or persistence; PK analyses confirmed robust expansion even under low tumor burden conditions. Previous studies in R/R B-ALL established positive correlations between clinical response rates and both peak CAR-T concentrations (C_max_) and area-under-the-curve (AUC_0-28d_) (15, 29, 30)-a relationship further validated by our findings. These results provide a mechanistic rationale for advancing CAR-T therapy into earlier-line and frontline treatment settings.

CD19 CAR-T cell therapy targets both malignant cells and normal CD19-expressing B cells, inducing BCA. Consequently, BCA serves as a surrogate biomarker for monitoring functional CAR-T persistence *in vivo* (10). Prior clinical evidence, including the ZUMA-3 trial, demonstrated that loss of peripheral blood BCA coincided with functional CAR-T clearance (15). Our study further establishes that BCA duration significantly correlates with *in vivo* CAR-T persistence (r=0.570, *p* < 0.001) and strongly predicts clinical efficacy. Patients with prolonged CAR-T persistence exhibited superior LFS. These results validate BCA as a robust activity biomarker and suggest that enhancing CAR-T persistence promotes durable disease control. While BCA serves as a functional marker of CAR-T functionality, its persistence requires close monitoring and proactive management of hypogammaglobulinemia to reduce infection risk. Although the rate of early infections in our cohort was manageable (18%), this study did not systematically capture late-onset infections, representing another notable limitation. The long-term infection risk associated with sustained BCA underscores the importance of regular assessment of immunoglobulin levels and timely immunoglobulin replacement therapy when indicated.

This study provides the first comprehensive evaluation of CD19 CAR-T cell therapy as earlier-line therapy for pediatric B-ALL patients with MRD positivity or chemotherapy intolerance. We demonstrate robust CAR-T expansion kinetics even in morphologically CR patients, resulting in high MRD clearance rates, durable responses, and manageable severe adverse events. These findings provide compelling evidence supporting earlier-line CAR-T implementation in high-risk pediatric ALL. Multicenter randomized controlled trials (RCTs) are warranted to validate this strategy’s clinical utility.

## Data Availability

The original contributions presented in the study are included in the article/**Supplementary Material**. Further inquiries can be directed to the corresponding authors.
