# Supplementary material for: Effective MRD clearance and long-term survival with CD19 CAR-T in pediatric B-ALL patients with MRD positivity or chemotherapy intolerance
Source: Front Immunol. 2025 Oct 6;16:1672509. doi: 10.3389/fimmu.2025.1672509 (PMC12536014; doi:10.3389/fimmu.2025.1672509)
Supplement: Supplementary file 1 [file Table1.docx]

| Supplemental Table 1. Patient and treatment characteristics among different subgroups | |
| --- | --- |
| Characteristic | N（%）/median（range） |
| Sex，male/female | 27（54%）/23（46%） |
| Age at diagnosis（y） | 7（1-16） |
| Age at infusion（y） | 7（1-25） |
| Cytogenetic/molecular genetic risk groups |  |
| Low-risk | 9（18%） |
| Intermediate-risk | 29（58%） |
| High-risk | 12（24%） |
| Allo-HSCT history | 5（10%） |
| CAR-T history# | 2（4%） |
| Risk factor |  |
| MRD re-emergence | 41 |
| MRD-positive | 2 |
| chemotherapy intolerance | 7 |
| Pre-LD MRD(%) | 0.03（0-3.22） |
| Post-LD MRD(%) | 0.02（0-2.06） |
| Time from diagnosis to CAR-T infusion（m） | 15.8（1.5-132.0） |
| CAR-T cell dose（×10^6^cells/kg） | 3.68(0.05-6.33) |

Supplemental Table 2. Overall survival (OS) and leukemia-free survival (LFS) among different subgroups

| Variables | N | 5y-OS,%(SE) | *p* | 5y-LFS,%(SE) | *p* |
| --- | --- | --- | --- | --- | --- |
| Age,y |  |  | 0.857 |  | 0.337 |
| 0-10 | 29 | 74.3±8.4 |  | 72.4±8.3 |  |
| ＞10 | 21 | 71.4±9.9 |  | 61.9±10.6 |  |
| Sex |  |  | 0.357 |  | 0.020 |
| Male | 27 | 66.7±9.1 |  | 54.1±9.8 |  |
| Female | 23 | 86.7±7.1 |  | 78.3±8.6 |  |
| Cytogenetic/molecular genetic risk groups |  |  |  |  |  |
| Low/ Intermediate -risk | 38 | 71.1±7.4 | 0.867 | 64.9±7.9 | 0.329 |
| High-risk | 12 | 81.8±11.6 |  | 75.0±12.5 |  |
| Risk factor |  |  |  |  |  |
| MRD re-emergence/MRD-positive | 43 | 68.9±7.0 | 0.449 | 66.1±7.4 | 0.356 |
| chemotherapy intolerance | 7 | 85.7±13.2 |  | 85.7±13.2 |  |
| Allo-HSCT  history |  |  | 0.575 |  | 0.665 |
| Yes | 5 | 75.0±21.7 |  | 60.0±21.9 |  |
| No | 45 | 73.3±6.6 |  | 68.2±7.0 |  |
| CAR-T history |  |  | 0.010 |  | 0.337 |
| Yes | 2 | 0 |  | 0 |  |
| No | 48 | 75.0±6.3 |  | 70.2±6.7 |  |
| Pre-LD MRD |  |  | 0.943 |  | 0.461 |
| ＜10^-4^ | 14 | 77.1±11.7 |  | 71.4±12.1 |  |
| ≥10^-4^ | 36 | 72.2±7.5 |  | 65.9±8.0 |  |
| Pre-LD MRD |  |  | 0.406 |  | 0.505 |
| ＜10^-3^ | 29 | 75.9±7.9 |  | 71.3±8.6 |  |
| ≥10^-3^ | 21 | 66.7±10.3 |  | 65.5±10.6 |  |
| Pre-LD MRD |  |  | 0.694 |  | 0.607 |
| ＜10^-2^ | 45 | 71.1±6.8 |  | 67.7±7.1 |  |
| ≥10^-2^ | 5 | 80.0±17.9 |  | 80.0±17.9 |  |
| Post-LD MRD |  |  | 0.369 |  | 0.981 |
| ＜10^-4^ | 23 | 68.1±10.0 |  | 65.2±9.9 |  |
| ≥10^-4^ | 27 | 77.8±8.0 |  | 69.4±9.0 |  |
| Post-LD MRD |  |  | 0.365 |  | 0.435 |
| ＜10^-3^ | 33 | 75.8±7.5 |  | 71.8±8.0 |  |
| ≥10^-3^ | 17 | 64.7±11.6 |  | 63.0±12.0 |  |
| Post-LD MRD |  |  | 0.701 |  | 0.834 |
| ＜10^-2^ | 47 | 72.3±6.5 |  | 69.1±6.9 |  |
| ≥10^-2^ | 3 | 66.7±27.2 |  | 66.7±27.2 |  |
| CAR-T dose |  |  | 0.989 |  | 0.715 |
| ≥3.68×10^6^  cells/kg | 25 | 72.0±9.0 |  | 70.0±9.5 |  |
| ＜3.68×10^6^  cells/kg | 25 | 72.0±9.0 |  | 67.8±9.4 |  |
| CRS |  |  | 0.080 |  | 0.466 |
| 0-2 | 42 | 76.2±6.6 |  | 70.8±7.1 |  |
| 3-4 | 8 | 58.3±18.6 |  | 50.0±17.7 |  |
| ICANS |  |  | 0.014 |  | 0.317 |
| No | 45 | 75.6±6.4 |  | 70.5±6.9 |  |
| Yes | 5 | 53.3±24.8 |  | 40.0±21.9 |  |
| Grade 4  neutropenia |  |  | 0.194 |  | 0.720 |
| Yes | 24 | 65.1±10.0 |  | 62.5±9.9 |  |
| No | 26 | 80.8±7.7 |  | 72.4±8.9 |  |
| MRD-CR on Day 28 |  |  | 0.409 |  | 0.003 |
| Yes | 48 | 71.9±6.6 |  | 70.8±6.6 |  |
| No | 2 | 100 |  | 0 |  |
| HSCT after  CAR-T |  |  | 0.178 |  | 0.266 |
| Yes | 32 | 65.6±8.4 |  | 63.9±8.8 |  |
| No | 18 | 83.3±8.8 |  | 77.8±9.8 |  |

Supplemental Table 3. Toxicity frequency including CRS, ICANS, neutropenia, and infections

| CRS |  |
| --- | --- |
| N | 16 |
| Any grade | 34 |
| Severe（≥Grade 3）CRS | 8 |
| ICANS |  |
| N | 45 |
| Any grade | 5 |
| Patients receiving tocilizumab | 14 |
| Patients receiving steroids | 7 |
| Infections within 30 days post-infusion | 9 |
| Neutropenia following infusion | 48 |
| The median nadir absolute neutrophil count（range）(×10^9^/L) | 0.53（0.05-1.45） |
| Duration of grade 4 neutropenia, median  (range) (d) | 8（1-28） |
| IL-6 elevation | 39 |
| N | 39 |
| Peak IL-6, median(range)( pg/mL) | 33.3（7.6-＞5000） |
| Time to peak(range) (d) | 8（1-22） |
| CRP elevation |  |
| N | 18 |
| Peak CRP, median(range)(mg/dL) | 22.4（8.8-108） |
| Time to peak(range) (d) | 9.5（2-17） |

Supplemental Table 4. Univariate analysis for ICANS

| Covariate | OR(95%CI) | *p* |
| --- | --- | --- |
| Pre-LD MRD＜10^-3^ | 0.356（0.240-0.527） | 0.010 |
| Post-LD MRD＜10^-3^ | 0.267（0.164-0.433） | 0.003 |
| No Grade 4 neutropenia | 0.556（0.322-0.960） | 0.182 |


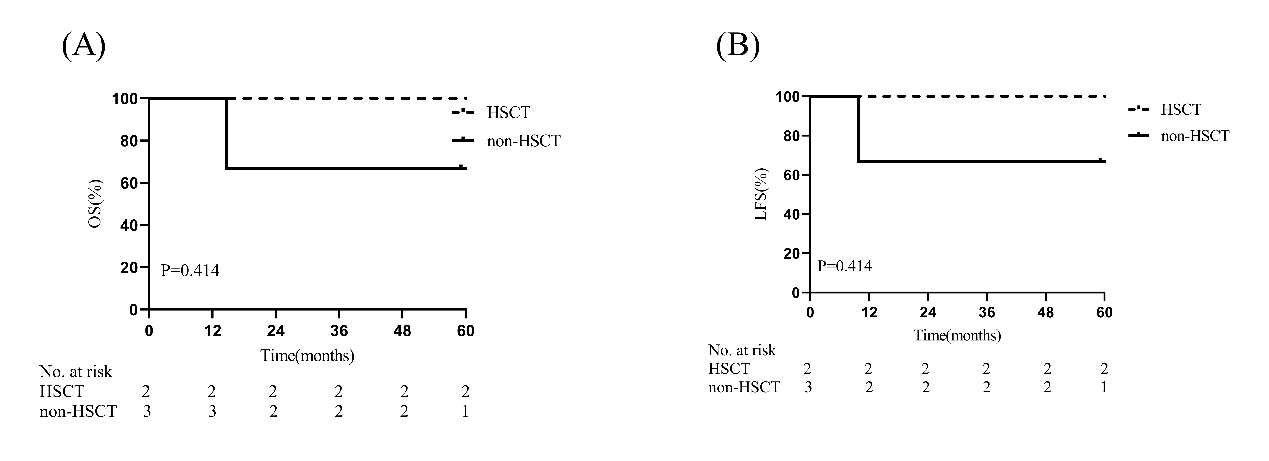


Supplemental Figure 1. Kaplan-Meier estimates of 5-year outcomes in the HSCT and non-HSCT arms in

MRD≥10^-2^. (A) OS (B) LFS.


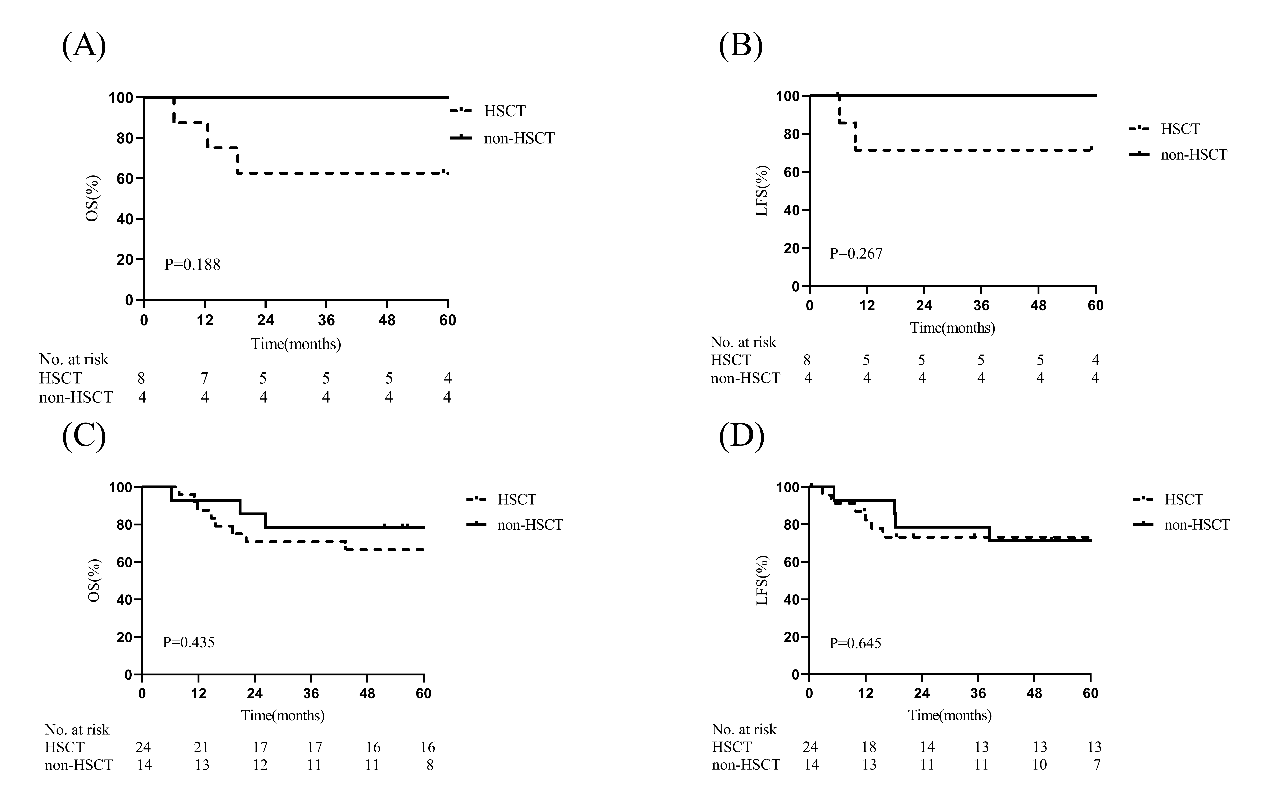


Supplemental Figure 2. Kaplan-Meier estimates of 5-year outcomes in the HSCT and non-HSCT arms in different genetic risk groups. (A) OS in high-risk group, (B) LFS in high-risk group, (C) OS in low/intermediate-risk group, (D) LFS in low/intermediate-risk group.


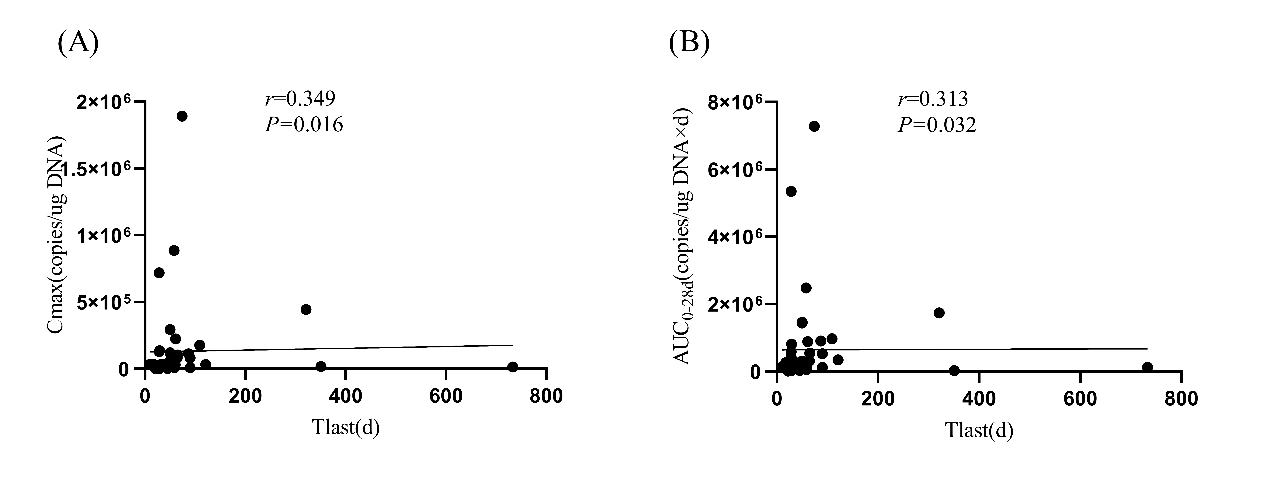


Supplemental Figure 3. Correlation between CAR-T cell expansion parametersand *in vivo* persistence (T_last_). (A) C_max_, (B)AUC_0-28d_.


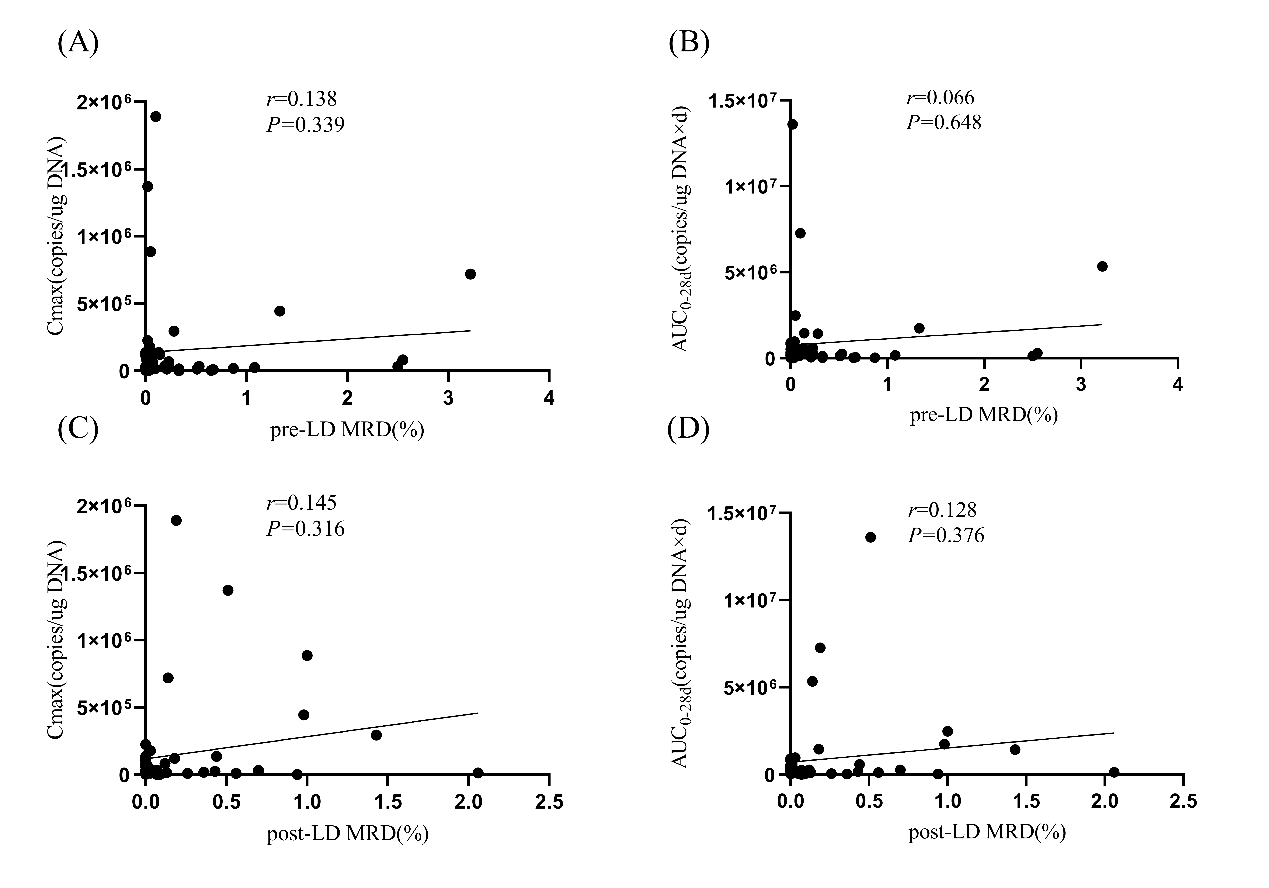


Supplemental Figure 4. Association between pre-infusion tumor burden and CD19 CAR-T *in vivo* expansion. (A) pre-LD MRD and C_max_, (B) pre-LD MRD and AUC_0-28d_, (C) post-LD MRD and C_max_, (D) post-LD MRD and AUC_0-28d_.
